# The Overview on the Pharmacokinetic and Pharmacodynamic Interactions of Triazoles

**DOI:** 10.3390/pharmaceutics13111961

**Published:** 2021-11-19

**Authors:** Andrzej Czyrski, Matylda Resztak, Paweł Świderski, Jan Brylak, Franciszek K. Główka

**Affiliations:** 1 Department of Physical Pharmacy and Pharmacokinetics, Poznan University of Medical Sciences, 6 Święcickiego Street, 60-781 Poznań, Poland; mresztak@ump.edu.pl (M.R.); glowka@ump.edu.pl (F.K.G.); 2 Department of Forensic Medicine, Poznan University of Medical Sciences, 6 Święcickiego Street, 60-781 Poznań, Poland; pswiderski@ump.edu.pl; 3 Department of Pediatric Gastroenterology and Metabolic Diseases, Poznan University of Medical Sciences, 27/33 Szpitalna Street, 60-572 Poznań, Poland; j.brylak@wp.pl

**Keywords:** drug-drug interaction, drug-food interaction, itraconazole, voriconazole, ketoconazole, isavuconazole, COVID-19

## Abstract

Second generation triazoles are widely used as first-line drugs for the treatment of invasive fungal infections, including aspergillosis and candidiasis. This class, along with itraconazole, voriconazole, posaconazole, and isavuconazole, is characterized by a broad range of activity, however, individual drugs vary considerably in safety, tolerability, pharmacokinetics profiles, and interactions with concomitant medications. The interaction may be encountered on the absorption, distribution, metabolism, and elimination (ADME) step. All triazoles as inhibitors or substrates of CYP isoenzymes can often interact with many drugs, which may result in the change of the activity of the drug and cause serious side effects. Drugs of this class should be used with caution with other agents, and an understanding of their pharmacokinetic profile, safety, and drug-drug interaction profiles is important to provide effective antifungal therapy. The manuscript reviews significant drug interactions of azoles with other medications, as well as with food. The PubMed and Google Scholar bases were searched to collect the literature data. The interactions with anticonvulsants, antibiotics, statins, kinase inhibitors, proton pump inhibitors, non-nucleoside reverse transcriptase inhibitors, opioid analgesics, benzodiazepines, cardiac glycosides, nonsteroidal anti-inflammatory drugs, immunosuppressants, antipsychotics, corticosteroids, biguanides, and anticoagulants are presented. We also paid attention to possible interactions with drugs during experimental therapies for the treatment of COVID-19.

## 1. Introduction

Invasive fungal infections (IFI) are a serious clinical problem. They are the result of a lack of immunity. They occur in critically ill patients in intensive care units, patients with severe hematological diseases, or patients after receiving chemotherapy in hematopoietic stem cell transplantation (HSCT) therapy, or HIV patients [1]. The following fungi species are responsible for severe fungal infections: *Aspergillus*, *Candida*, *Scedosporium*, *Mucorales*, *Cryptococcus*, and *Candida*. However, the most common IFI’s are those caused by *Aspergillus* and *Candida*. The mortality for aspergillosis is 56%, and candidiasis is 10–25%. Invasive aspergillosis is found in patients with acute myeloid leukemia, myelodysplastic syndrome, or allogeneic HSCT. The other groups are patients with liver cirrhosis or chronic obstructive pulmonary disease [2,3,4,5]. Antifungal treatments started with an introduction of Amphotericin B deoxycholate in 1958, which became a criterion standard for treatment for more than 40 years [6]. It is a polyene drug obtained from the species of *Streptomyces*. However, due to its nephrotoxicity and other serious adverse effects, it was necessary to find other antifungal agents that provide safer therapy. Over the past three decades, a significant development was observed concerning antifungal treatment, such as new formulations of amphotericin, echinocandins, and azole derivatives. The last group comprises the aromatic compounds with imidazole and triazole rings. In 1979 and 1981, miconazole and ketoconazole were introduced as a therapy, respectively. Ketoconazole is an imidazole derivative that was initially administered for systemic use. However, it was superseded by the new generation of drugs, i.e., triazoles. Triazoles, also called the second generation of azoles, are represented by itraconazole, voriconazole, posaconazole, and isavuconazole [6,7]. Their activity is based on the inhibition of ergosterol synthesis in the fungal cell, required for membrane integrity and the function of membrane associated proteins. They are characterized by a broad antifungal activity [8,9,10].

The antifungal agents are often coadministered with other drugs—this may lead to pharmacokinetic and pharmacodynamic interactions. The interaction may be encountered on the absorption, distribution, metabolism, and elimination (ADME) step. The factor that limits absorption is interaction with food. It can be a two-way activity—the meal may restrict or enhance bioavailability. The other factor is the glycoprotein-P (P-gp) and cytochromes present in the intestines, which may limit absorption. Itraconazole and posaconazole are strong inhibitors of CYP3A4. Voriconazole has a moderate activity towards this enzyme as well as CYP2C9. However, it is a strong inhibitor of CYP2C19 and also its substrate. Itraconazole is the substrate for CYP3A4. They have a strong affinity towards these enzymes [6]. The interaction in the metabolism step with the interaction with CYP enzymes may increase or decrease exposure on the drug. This may result in a change of activity of the drug, from lacking any to serious side effects.

The purpose of this review is to discuss the studies on treatment with antifungal agents with relevance to their pharmacokinetic interaction. The paper analyses the interactions with other drugs, and the impact of the fed state on the representatives of the second generation of antifungal drugs, i.e., itraconazole, voriconazole, posaconazole, and isavuconazole. The PubMed and Google Scholar base were searched to collect literature data. There results are reported in both regular clinical trials and case studies.

## 2. Itraconazole

Itraconazole is an antifungal agent with a broad spectrum of antifungal activity [11]. It can be administered through *iv* and *po* administration. Itraconazole can be administered once or twice a day. The single dose can be 100–200 mg. It should not exceed 400 mg daily. The duration of the treatment depends on the type of infection—it may take 1 to 12 weeks. The elimination half-life is 20 h after a single dose of 200 mg. It stays up to 30 h in a steady-state condition. It is caused by the saturation of the metabolism [12,13]

### 2.1. The Impact of Food and pH in the Gastrointestinal Tract on Absorption of Itraconazole

The impact of food on itraconazole pharmacokinetics is unpredictable. Zimmerman et al. [14] found that food has an unpredictable effect on itraconazole absorption. The consumption of heavy breakfast delayed the t_max_ by two hours. The coefficient of variation for AUC_0-72_ was 62% after a meal, and the relative bioavailability ranged from 0.35 to 3.74, and C_max_ ratios for postprandial vs. fasting of 0.27–5.71. However, the absorption of itraconazole is enhanced by a low stomach pH, high-fat content, and long gastric retention time [15]. The investigation led by Barone et al. and van Peer et al. proved that itraconazole should be taken with food or shortly after meals to provide optimal oral-systemic availability [16,17].

Jaruratansirikul et al. investigated the impact of omeprazole on the pharmacokinetics of itraconazole [18]. The concomitant administration of the fungal agent with omeprazole resulted in a lower concentration of itraconazole. The C_max_ and AUC_0-24_ were reduced by 66% and 64%, respectively. A slight increase of t_max_ by 27% was observed for the group that also took omeprazole. However, it was not a statistically significant change. The study implies that an acidic pH is required for complete dissolution and absorption. When the concomitant use of omeprazole and itraconazole cannot be avoided, an increase in the itraconazole dose should be considered. These findings are from the data from the former study of Jaruratansirikul et al. concerning the impact of acidic beverages on the absorption of itraconazole [19]. Coca-Cola enhanced the bioavailability of the drug. This might have been caused by the calories contained in the beverage, which delayed gastric emptying and stimulated hepatic blood flow. It led to an increase of C_max_, t_max_, and AUC by 2.21, 1.32, and 1.80-fold, respectively.

### 2.2. Interaction with CYPs

The interaction is mainly concerned with CYP3A4. Itraconazole is an inhibitor of CYP3A4 and is also metabolized by this enzyme. The main metabolites are hydroxyitraconazole, keto-itraconazole, and N-desalkylitraconazole. Hydroxyitraconazole and ketoitraconazole are also the substrates of CYP3A4 [20]. The IC_50_ of itraconazole against CYP3A4 is 0.0326 µM. The IC_50_ against CYP2C9 and 2C19 is above 10 µM [21]. The examples of the interactions are listed below.

#### 2.2.1. Ibrutinib

Tapaninen et al. investigated the drug-drug interactions (DDI) between itraconazole and ibrutinib. Ibrutinib is a Bruton’s tyrosine kinase inhibitor that is characterized by extensive first-pass metabolism. Its bioavailability is 3% and it is metabolized by CYP3A4 [22]. Itraconazole is a strong inhibitor of CYP3A4, and the potential interaction may increase the bioavailability of ibrutinib. The concomitant administration of itraconazole with ibrutinib resulted in a 10-fold increase in the geometric mean value of AUC_0-∞_ and an 8.8-fold increase in C_max_ for ibrutinib. This DDI can be considered useful for several reasons. Therefore, a smaller dose of ibrutinib can be used when itraconazole is given. The information given in the summary product characteristics shows that the dose of ibrutinib should be reduced up to 280 mg or 140 mg once daily when coadministered with moderate or strong CYP3A4 inhibitors, respectively, or should even be withheld [23]. The authors suggest that the decreased dose of ibrutinib (140 mg) is too high, and it should be ca. one-tenth of the regular treatment dose, which is 420 mg or 560 mg. It also reduces the costs of the therapy. Itraconazole also reduces interindividual variability in exposure to ibrutinib, which makes the pharmacokinetics more predictable [22].

#### 2.2.2. Efavirenz

Resistant fungal infections can occur in patients with HIV. HIV treatment involves the administration of efavirenz, which is an inducer of CYP3A4 [24]. Itraconazole is not only the inhibitor, but also is the substrate for CYP3A4. Kaewpoowat et al. investigated the impact of efavirenz on the pharmacokinetics of itraconazole and its metabolite (hydroxyitraconazole). The study proved that the simultaneous administration of efavirenz and itraconazole led to the decreased steady-state concentration of itraconazole and its metabolite. Exposure of itraconazole and its metabolite in people with HIV, receiving 200 mg capsule twice a day, was lower than in healthy volunteers. The reduction of the exposure was significant; however, it must be further evaluated when the secondary prophylaxis is considered [25].

#### 2.2.3. Statins

As an inhibitor of CYP3A4, itraconazole can interact with statins that are metabolized by this pathway. Kantola et al. investigated the DDI between itraconazole and atorvastatin [26]. The C_max_ and t_max_ of atorvastatin acid were not altered by itraconazole. The t_max_ remained unchanged also for atorvastatin lactone. The AUC_0-72_ increased three-fold for acid and four-fold for lactone when coadministered with itraconazole. The peak serum concentration and half-life increased more than two-fold for atorvastatin lactone. The mean half-life also increased more than two-fold for atorvastatin acid. The main metabolite was 2-hydroxyatorvastatin (acid and lactone form). The C_max_ of 2-hydroxyatorvastatin acid and lactone was lower when atorvastatin was used concomitantly with itraconazole, suggesting the inhibition of the formation during the first pass metabolism. The t_max_ for 2-hydroxyatorvastatin acid was prolonged from 2 h to 11 h.

The prolongation of the half-life for atorvastatin acid and lactone might be a result of decreased systemic clearance. The gender-related differences were not observed. CYP3A4 is an enzyme for which other statins are the substrates—lovastatin and simvastatin. Pravastatin is removed from the human body practically unchanged. CYP2C9 metabolizes fluvastatin. Itraconazole does not impact the AUC of fluvastatin and pravastatinsignificantly. The concomitant intake of lovastatin and simvastatin with itraconazole resulted in up to a 20-fold increase of AUC for the hypolipidemic drug (its acid form). The concomitant use of atorvastatin and itraconazole should be avoided due to the risk of myopathy, which results from the inhibition of the biotransformation of atorvastatin [26,27].

#### 2.2.4. Oxycodone

Saari et al. investigated the impact of itraconazole on oxycodone, the analgesic agent, and the relevance of the administration [28]. Oxycodone is a semi-synthetic µ-opioid agonist used in the treatment of acute pain in cancer patients. The predominant metabolic pathway is N-demethylation mediated with CYP3A4. CYP2D6 is the other enzyme that is involved in the metabolism of oxycodone. Itraconazole reduced the plasma clearance by 32%. It increased the half-life after intravenous and oral administration of oxycodone from 3.8 to 5.5 h, and from 4.0 to 5.9 h, respectively. The values for the volume of distribution were unchanged. The prolongation of the half-life was a result of the decrease in clearance. The oral administration of oxycodone resulted in a 1.4-fold increase of C_max_ and a 2.4-fold increase in AUC_0-∞_. Itraconazole inhibited both the gut and the liver CYP3A4. It is one of the reasons for the higher increase of AUC_0-∞_ for oral intake than for *iv* administration (2.4 vs. 1.5). However, this can also be related to the inhibition of P-gp. For oral administration of oxycodone, an increase of AUC_0-∞_ by 144% is observed. The changes for the pharmacokinetic parameters were observed in the elimination phase. The peak concentration was increased by 45%. The changes also concerned the metabolites. The AUC_0-48_ of noroxycodone decreased by 49% and for oxymorphone increased by 359% after oral administration. This study proved that dose adjustment might be necessary during the coadministration of itraconazole with oxycodone. The inhibition of N-demethylation via CYP3A4 by itraconazole may result in opioid toxicity due to the increased bioavailability [28].

#### 2.2.5. Midazolam

A serious interaction was observed for midazolam. Olkkola et al. investigated the impact of itraconazole on the midazolam, a sedative agent [29]. Midazolam is characterized by the intensive first-pass effect and the relatively low bioavailability. After administration of itraconazole, the increase in the half-life of midazolam was observed from 2.8 h to 7.9 h. The inhibited elimination resulted in a 10.8-fold increase of AUC_0-∞_, a 3.4-fold increase of C_max,_ and a 1.5-fold increase of t_max_. This interaction resulted from the inhibition of the CYP 3A cytochrome, for which midazolam is the substrate and itraconazole is an inhibitor. This interaction resulted in profound sedative effects. The volunteers could hardly be awakened during the first hour after taking 7.5 mg of midazolam, and most of them experienced amnesia for a couple of hours. The differences in the results for the psychomotor tests (the digit symbol substitution test or the Maddox test) were statistically significant after 6 h of administration of midazolam. The coadministration of midazolam and itraconazole should be avoided, or the dose of prescribed midazolam should be reduced [29].

#### 2.2.6. Alprazolam

Alprazolam is the other drug which activity is pointed at the central nervous system. CYP3A4 metabolizes it, and its metabolites are hydroxylated derivatives. Yasui et al. [30] investigated the impact of itraconazole on the pharmacokinetics of alprazolam. Itraconazole did not affect the C_max_ and t_max_ of alprazolam. However, prolongation of the elimination phase was observed. For the itraconazole phase, the clearance of alprazolam decreased by ca. 2.5-fold and the half-life increased ca. 2.7-fold. The AUC_0-48_ and AUC_0-∞_ increased by 1.6 and 2.7-fold, respectively. The prolongation in the elimination phase led to a change in psychomotor functions. The extent of the depression was proportional to the concentration of alprazolam in the plasma, which implies that itraconazole enhanced the sedative effect due to the prolongation of the elimination phase and elevated plasma concentration of the sedative [30].

#### 2.2.7. Haloperidol

Haloperidol is a drug that CYP2D6 and CYP3A4 intensively metabolize. CYP3A4 plays a vital role in N-dealkylation. CYP2D6 is highly polymorphic, and the activity may be dependent on the ethnic group. Park et al. [31] investigated the relevance of the CYP2D6 genetic polymorphism on the haloperidol pharmacokinetics and pharmacodynamics when itraconazole (a potential CYP3A4 inhibitor) was coadministered. The study showed that itraconazole significantly increased AUC_last_ and AUC_inf_ for both genetic types, i.e., CYP2D6*10/*10 and CYP2D6*1/*1. The general increase was by 81% for AUC_inf_ when compared to the placebo group. The presence of the CYP2D6*10/*10 allele led to a two-fold increase in the AUC_inf_ of haloperidol. However, for the subjects with both the CYP2D6*10/*10 allele and itraconazole pretreatment, the observed decrease of the oral clearance of haloperidol was 42% of the subjects of genotype CYP2D6*1/*1 for the placebo phase. The analysis of the pharmacodynamic effects showed that subjects with CYP2D6*10/*10 after pretreatment of itraconazole had higher scores of BARS (Barnes Akathisia Rating Scale) than the group with CYP2D6*1/*1 and placebo, but it was not statistically significant. This study proved that, as a CYP3A4 inhibitor, itraconazole could also augment the effect of CYP2D6*10 on the pharmacokinetics and pharmacodynamics of haloperidol.

#### 2.2.8. Phenytoin

Ducharme et al. observed the interaction between itraconazole and phenytoin. The concomitant administration of these drugs led to a decreased AUC of itraconazole by 90% and a 15-fold increase in clearance. The maximum peak concentration and half-life were reduced up to ca. 20% of the control values. The changes in the pharmacokinetic parameters for itraconazole were similar to those for hydroxyitraconazole. The multiple doses of itraconazole resulted in a 10% statistically significant increase of AUC for phenytoin. It was caused by the fact that phenytoin was metabolized by CYP2C9, and the transformation might be inhibited with itraconazole. These findings may explain the failure of the antifungal treatment in the patients receiving phenytoin [32].

### 2.3. Interaction with P-gp

P-gp is an ATP-dependent plasma transporter. It is present in the cellular membranes of the gastrointestinal tract, liver, and kidneys [33]. It is responsible for removing xenobiotics from the human body. Tapaninen et al. [34] conducted a study where itraconazole was coadministered with aliskiren, a drug that has an affinity to P-gp. The result of the interaction was a 5.8-fold increase in C_max_ and 6.5-fold increase in the AUC of aliskiren. This change is attributed mainly to the inhibition of P-gp by itraconazole. In the study conducted by Lempers et al., the IC_50_ of P-gp for itraconazole was 2 µM [35].

### 2.4. Conclusions

Despite the unpredictible impact of food on absorption, itraconazole should be taken with food. The concomitant use of agents can change the pH and may influence the bioavailability. The calories from food and beverage stimulate blood flow and delay gastric emptying. Itraconazole interactions with CYP concern mainly CYP3A4. The inhibition of the enzyme may result in a serious side effects, especially for the drugs that are targeted at the central nervous system. It should be also cautiously used with drugs that have an affinity to P = gp.

The interactions are listed in Table 1.

## 3. Voriconazole

Voriconazole is a triazole antifungal agent with a broad spectrum of fungicidal activities, including *Aspergillus*, *Candida*, *Scedosporium*, and *Fusarium* species [36]. It is used to treat severe fungal infection that may occur in patients with immunodeficiency as well as in intensive care unit patients. It is available in intravenous and oral formulations. The total dose for patients with body weight >40 kg is 200 mg twice a day. The patients with a body weight below 40 kg receive 100 mg twice a day. The treatment starts with a loading dose twice the amount of the daily dosage [37]. At higher doses, voriconazole represents non-linear pharmacokinetics—due to its capacity–limited elimination. Multiple-dose studies showed a disproportionate increase in AUC and C_max_ with doses of both intravenous and oral formulations [38]. The in vitro tests proved that voriconazole is neither an inhibitor nor a substrate for P-gp [33,35].

### 3.1. Impact of Food and pH in the Gastrointestinal Tract on the Absorption of Voriconazole

Purkins et al. [39] conducted a study that evaluated the effect of food on the pharmacokinetic parameters of voriconazole. Voriconazole was administered twice a day in the fasted and fed state. The pharmacokinetics analysis showed that the coadministration of voriconazole with food led to a decrease of C_max_ and AUC as a result of reduced absorption. In the fed state, t_max_ was longer when compared with the fasted state. The elimination remained unchanged for both groups. The total bioavailability was reduced by 22%. The study proved that voriconazole should not be administered with a meal or immediately following a meal.

Food is not the only agent that may modify the bioavailability of the drugs. The absorption of the drug also depends on the pH of GI-tract. Proton pump inhibitors are widely used to treat oesophageal reflux, and gastric and duodenal ulcers. Their activity is based on the inhibition of the secretion of gastric acid. Omeprazole, rabeprazole, esomeprazole, and pantoprazole are competitive inhibitors of CYP2C9, whose activities were proven in the in vitro tests to make the interaction with voriconazole possible. Heinz et al. analyzed the plasma through concentrations of voriconazole in patients who received ranitidine and pantoprazole. The patients who received ranitidine had lower voriconazole trough concentrations, contrary to the ones with the comedication of pantoprazole [40]. Wood et al. [36] determined the impact of omeprazole on the pharmacokinetics of voriconazole in steady-state. The loading dose of voriconazole was 400 mg BID and the maintaining dose was 200 mg. The coadministration of omeprazole had little effect on the AUC_τ_ of voriconazole on day 1. At steady-state on day 10, the C_max_ and AUC_τ_ increased by 15% and 41%, respectively. T_max_ remained unchanged. The interindividual variability of these changes was not considered to be clinically relevant, and the combination of omeprazole and voriconazole was well tolerated. A significant increase in the voriconazole concentration was also observed for pantoprazole, esomeprazole, and rabeprazole [41,42]. When coadministered with omeprazole or pantoprazole (CYP inhibitors), the increase in the minimum voriconazole concentration was also proven in a retrospective study conducted by Cojutti et al. [43]. In the study, the interaction with both the type of administration and the dose of PPI was estimated. The lowest impact was noted for 20 mg pantoprazole per *os,* and the highest was observed for 80 mg pantoprazole *iv*. These findings differ from the in vitro study where omeprazole was the most potent inhibitor [41]. This difference might be caused by incomplete bioavailability, which has a mild effect on the C_min_ voriconazole. A significant impact was observed for PPI administered *iv* at high doses—the trough concentration of voriconazole was significantly increased. Clinicians should be aware that simultaneous *iv* coadministration of PPI in doses ≥40 mg may result in the dose of voriconazole needing adjustment [43].

### 3.2. The Interaction with CYPs

Voriconazole is metabolized mainly via CYP2C19. This enzyme has eight variant alleles from CYP2C19*1 to CYP2C19*8. For the type *1/*1, the normal activity of the enzyme is observed—they are extensive/normal metabolizers (EM or NM). Intermediate metabolizer (IM), poor metabolizer (PM), rapid metabolizer (RM), and ultra-rapid metabolizer (UM) are observed for the following genotypes *1/*2, *2/*2, *1/*17, and *17/*17, respectively [44]. In addition, 2–5% of Caucasians and 15–20% of Japanese subjects are functionally absent or deficient of CYP2C19. In the in vitro study, the involvement of CYP2C9 in voriconazole metabolism is limited. The data imply that voriconazole has a 50-fold lower affinity to CYP3A4 than CYP2C19. However, because of the lack of CYP2C19 pathway in the poor metabolizers, the alternative metabolic track via CYP3A4 may become more important for voriconazole metabolism [45,46]. None or a reduced activity was observed for the remaining seven types (from *2 to *8). The main defective alleles were CYP2C19*2 and CYP2C19*3. The former is responsible for 75–85% of poor metabolizers (PM) in Orientals and Caucasians. The latter is rare in Caucasian population, but is predominant in Oriental populations. The other enzymes are CYP2C9 and CYP3A4 [38,47,48,49]. Voriconazole is an inhibitor of CYP2C9, CYP2C19, and CYP3A4, and the IC_50_ values are on a similar level, and amount to 8.4 µM, 8.7 µM, and 10.5 µM, respectively [21,50]. The main metabolite is voriconazole N-oxide. The others are hydroxy-voriconazole and dihydroxyvoriconazole [51]. The pharmacokinetics is variable between healthy subjects and the concentration may vary by even 100-fold between patients. This is caused by the polymorphism of CYP2C19. The polymorphism of the other enzymes such as CYP3A4 and 2C9 are not considered as being clinically relevant. The affinity to CYP3A4 is 50-fold lower when compared with the CYP2C19. Only a small fraction of the drug is metabolized with 2C9 [46,52,53]. The examples of the interactions are listed below.

#### 3.2.1. Ritonavir

The coadministration of voriconazole with ritonavir, an inhibitor of CYP3A4, led to increased exposure to voriconazole [45]. The coadministration of ritonavir caused the increase in the following pharmacokinetic parameters of voriconazole: C_max_, AUC_0-48,_ AUC_0-∞_, half-life, MRT, and the amount excreted in the urine. The decrease was observed in nonrenal and oral clearance. The renal clearance remained unchanged, and the volume of distribution was slightly reduced. The analysis towards the CYP2C19 alleles showed that the concomitant administration of ritonavir in homozygous EM led to an increase in the following pharmacokinetic parameters: AUC_0-48_, AUC_0-__∞_, MRT, and the amount excreted in the urine. In heterozygous EM, an additional increase was noted for the following parameters: half-life and C_max_. The decrease was noted for Cl_oral_, Cl_nonrenal_ for homo- and heterozygous EM. The volume of distribution was reduced in homozygous EM. The differences became more significant in PM. The half-life was prolonged. The oral and nonrenal clearance was significantly reduced when compared with the control group. However, the renal clearance was not affected by ritonavir in PM, but the observed values were lower than for both homozygous and heterozygous EM. The study explained the wide intersubject variability in voriconazole treatment. Ritonavir application, of the potent CYP3A4 inhibitor, resulted in a 42% reduction of the apparent oral clearance. However, the possibility of interaction with the CYP3A4 inhibitor is not obvious—interactions with azithromycin, which was a weak inhibitor, and gentamycin, a potent inhibitor, were not observed [54,55]. The patients with a CYP2C19 deficiency should be aware of more frequent side effects resulting from the elevated voriconazole levels. CYP3A4 becomes an important metabolic pathway in individuals with a low CYP2C19 function.

#### 3.2.2. Non-Steroidal Anti-Inflammatory Drugs

Non-steroidal anti-inflammatory drugs (NSAIDs) are widely used. CYP2C9 metabolizes them, and coadministration with the inhibitor of the enzyme increases the risk of interaction. Hynninen et al. [56] conducted a study where the evaluation of the impact of voriconazole on the pharmacokinetics of S-(+) and R-(−)- ibuprofen was done. Voriconazole significantly increased the plasma levels of S-(+)-ibuprofen, resulting in increased AUC by 105% and C_max_ by 22%. The elimination was prolonged and the half-life increased by 43%. The difference in t_max_ was not statistically significant. The impact of voriconazole on the pharmacokinetics of R-(−)-ibuprofen was lower—an increase by 20% was observed for AUC. The half-life was slightly shortened by 7%. C_max_ and t_max_ were not affected by the voriconazole pretreatment. The elevated level of S-(+) ibuprofen may increase cyclooxygenase 1 and cyclooxygenase 2 inhibition. It may reduce prostaglandin E2 and thromboxane B2 formation and lead to cardiovascular, renal, and gastrointestinal (acute bleeding) effects. The patients treated with voriconazole and high doses of ibuprofen should be observed [56,57,58]. The other NSAID that CYP2C9 intensively metabolizes is diclofenac. The other study conducted by Hynninen et al. [59] examined the impact of voriconazole on diclofenac’s pharmacokinetics. The coadministration of voriconazole led to an increase of both C_max_ and AUC of diclofenac. The maximum concentration of diclofenac increased two-fold, and the exposure to diclofenac was 178% higher when compared with the control group. Voriconazole also reduced the renal clearance of diclofenac by 47%. The genetic study conducted on the subjects proved that the variations in the CYP2C9*1*1 gene (wild type) such as CYP2C9*1*2 and CYP2C9*1*3 do not influence the diclofenac pharmacokinetics. The half-life and t_max_ were unaffected by voriconazole pretreatment. This study proved that the patients concomitantly receiving voriconazole and diclofenac should take lower doses for pain relief [59]. Li et al. [60] analyzed the impact of voriconazole on the following representatives of NSAIDs pharmacokinetics: ibuprofen, celecoxib, tenoxicam, and piroxicam. The predictions were made using physiologically based pharmacokinetic models (PBPK). To construct the PBPK model, the following parameters were used: partition-coefficient (logP), pK_a_, effective permeability, dose, formulation, molecular weight, blood-to plasma concentration, and unbound fraction. The applicability of the PBPK models was validated with the results of the study conducted by Hynninen et al. [59], which confirmed the predictive capability of the models tested by Li et al. [60]. The analysis of the predicted parameters for the tested representatives of the NSAIDs showed that C_max_ and AUC increased by 21% and 51%, respectively, for celecoxib administered with voriconazole. Voriconazole has a weak effect on the increase of AUC of ibuprofen (7%), tenoxicam (2%), and piroxicam (1%), as well as on C_max_, which was increased by 1% for these three drugs. The simulated pharmacokinetic parameters for ibuprofen differ from the ones in the previous study [56]. It was caused by the fact that in the study conducted by Hynninen et al. the changes of the concentrations for racemic ibuprofen administered orally were analyzed. Li et al. [60] took into consideration the data obtained after the intravenous administration of ibuprofen. Ibuprofen undergoes a first-pass effect, and the interaction might be not observed for parenteral administration [60].

#### 3.2.3. Imatinib

Antifungal drugs are commonly used during the treatment of tumors. The therapy of chronic myeloid leukemia and gastrointestinal stromal tumors often requires antifungal agents against invasive aspergillosis [61]. There are several studies in which the voriconazole effect on imatinib has been studied. In the study of Lin et al. [62], which was conducted on an animal model, the impact of voriconazole on the imatinib and its metabolite (GCP74588) was tested. The study proved that concomitant use with voriconazole resulted in a higher value of imatinib C_max_, which increased by 36.8%. The differences between the other analyzed parameters were not statistically significant. This implied that voriconazole inhibits CYP3A4 in a mild way [62]. In the case of the metabolite GCP74588, more changes are observed. C_max_, AUC_0-t_, and AUC_0-__∞_ were decreased by 55.8%, 49.9%, and 49.7%, respectively. An increase in the following parameters was observed: t_0.5_, V_z/F_, and Cl_z/F_ by 30.7%, 170%, and 110%, respectively. T_max_ and MRT remained unchanged. The impact of voriconazole on imatinib metabolism was also studied in vitro [63]. The inhibitory activity stretched not only on voriconazole, but also on the other representatives of azole drugs such as ketoconazole, itraconazole, posaconazole, and fluconazole. Imatinib is an inhibitor of CYP3A4 that may influence the pharmacokinetic parameters of voriconazole, which is also a substrate for this enzyme [64]. The in vitro studies proved that imatinib inhibited the metabolism of voriconazole in rat liver microsomes in a sensible way. It is reduced to 38.9%. On the other hand, voriconazole causes a lower inhibition of the imatinib metabolism—it is inhibited to 54.4%. The in vivo study on the animal model proved that voriconazole did not change the clearance and bioavailability of imatinib. Only the C_max_ of imatinib was increased by 18.8%. The AUC_0-t_, AUC_0-__∞_, and C_max_ of the imatinib metabolite (CGP74588) decreased. In voriconazole, AUC_0-t_, AUC_0-__∞_, MRT_0-__∞_, t_max_, and C_max_ increased contrary to AUC_0-t_, AUC_0-__∞_, and C_max_ of voriconazole N-oxide, which decreased significantly. The concomittant administration of imatinib and voriconazole led to both the increase of voriconazole concentration and decrease of voriconazole N-oxide formation. It confirmed the results from the in vitro study that imatinib inhibits the metabolism of voriconazole. The coadministration of imatinib with other CYP3A4 substrates with a narrow therapeutic window like voriconazole should be done with caution. Imatinib alters the profile of both voriconazole and voriconazole N-oxide [61].

#### 3.2.4. Phenytoin

The other drug that is an inducer of CYP3A4 is phenytoin. It is also a substrate and inducer for CYP2C9 and CYP2C19; however, the formation of the metabolite is mediated exclusively by CYP2C9 [65,66,67]. The concomitant administration of voriconazole and phenytoin could lead to elevated levels of phenytoin due to the inhibition of the metabolism. Phenytoin has a narrow therapeutic index (10–20 µg/mL), nonlinear pharmacokinetics (due to zero-order clearance), and unpredictable absorption. The interactions could result in side effects [67,68]. The repeated administration of phenytoin with voriconazole resulted in a 50% decrease in C_max_ and 70% decrease in AUC_τ_ of voriconazole when the repeated dose of the antifungal agent was 200 mg. It may lead to the failure of antifungal therapy. However, the change of the dose of voriconazole from 200 mg to 400 mg BID resulted in higher concentrations that were compensated for the effect—C_max_ and AUC_τ_ were restored. They increased by 34% and 39%, respectively, without apparent safety or tolerability implications. On the other hand, the repeated increased dose of 400 mg of voriconazole led to the increase of phenytoin C_max_ and AUC_τ_ by ca. 70% and 80%, respectively, compared with the placebo group, which may result in concentrations out of the therapeutic index [67]. The concentrations of these drugs need to be monitored because of the non-linear pharmacokinetics, especially when they are concomitantly used.

#### 3.2.5. St. John’s Wort

The other drugs that may interact with antifungal therapy are herbal ones. Herbs are widely used and often regarded as safe. They are easily available on the market. St John’s wort is recommended by Traditional Chinese Medicine practitioners and is widely prescribed for depression in European countries. Its coadministration with such drugs as cyclosporine, oral contraceptives, digoxin, indinavir, tacrolimus, imatinib, indinavir, and warfarin leads to a decrease in the plasma concentration, which results in a treatment failure. St John’s wort is a potent inducer of CYP3A4, and it increases the possibility of interaction with drugs that undergo metabolism via this enzyme. The in vitro study on hepatic cell cultures showed that hyperforin, a substance produced by St. John’s Wort, induced CYP3A4 and CYP2C19 upon chronic exposure. On the other hand, after single exposure at high concentrations, hyperforin inhibits CYP3A4, CYP2C9, and CYP2C19, but the inductive effect predominates with chronic exposure [69,70,71,72,73,74]. The coadministration of St. John’s wort with voriconazole led to changes in the pharmacokinetic parameters. On the first day of the concomitant intake with voriconazole, an increase of AUC_0-10_ by 22% was observed, but after 15 days of daily administration of St. John’s wort, an extensive decrease of AUC_0-10_ by 43% was observed. A similar trend was observed for AUC_0-∞_ and C_max_. The AUC_0-∞_ and Cl/F on the first day were not changed. On day 15, AUC_0-∞_ was reduced by 59%, and Cl/F increased from 390 to 952 mL/min. Renal clearance increased from 1.60 to 2.20 mL/min. It was reduced only on day 1. These results imply that the effect on the first day is limited only to the absorption phase of voriconazole [70]. The half-life shortened on day 1 and further shortened on day 15. When the genotypes of CYP2C19 were taken into consideration, it was found that AUC_0-10_ and AUC_0-∞_ did not change on day 1, but significantly decreased on day 15 in the CYP2C19 wild type group and CYP2C19*1*2 group and two participants of CYP2C19*2*2 group. In Cl/F, a significant change was not observed for the control group compared with day 1. A significant increase was observed on day 15 for the wild-type and CYP2C19*1*2 and for the two subjects in the CYP2C19*2*2 group. The increase in Cl/F was the most significant for the wild-type group compared with CYP2C19*1*2. However, the relative increase for these two groups was similar. The corresponding changes were also observed for AUC_0-__∞_—similar relative values for reduction were observed for these two groups [70].

#### 3.2.6. Simvastatin

The previously mentioned interactions took into consideration CYP2C19 as a main metabolic pathway for voriconazole metabolism. The drug that is metabolized via CYP3A4 is simvastatin. The inhibition of statins metabolism may lead to renal and hepatic dysfunction and hazardous muscle toxicity [75]. Doran et al. [76] described a patient where the interaction between voriconazole and simvastatin was observed. Blood tests showed that the hepatic enzyme levels were highly elevated. After cessation of the coadministration of these two drugs the results of the blood tests improved. The patient died of respiratory failure secondary to respiratory muscle weakness on day 10 after the concomitant therapy was stopped.

#### 3.2.7. Rifampicin

The other significant drug–drug interaction for voriconazole is the interaction with rifampicin [77], an inducer of CYP3A4 cytochrome [78]. The clinical microbiologists advised introducing rifampicin to the therapy on day 7 of the antifungal treatment. After 30 days of rifampicin therapy, the C_max_ of voriconazole decreased by 99% from initial 3.92 µg/mL to 0.038 µg/mL. Exposure to the antifungal agent was also reduced—the AUC_0-12_ changed from 27.4 h×µg/mL to 0.145 h×µg/mL. The induced metabolism of voriconazole led to an increase of the metabolic ratios in the plasma for each metabolite. A 45-fold increase was observed for N-oxide, 178-fold was observed for hydroxyvoriconazole, and 422-fold increase for dihydroxyvoriconazole, leading to a lack of a fungicidal effect.

#### 3.2.8. Glucocorticoids

The other factor influencing voriconazole concentration is coadministration with glucocorticoid (prednisone/prednisolone, methylprednisolone, and dexamethasone). The analysis performed by Dolton et al. [42] showed that glucocorticoids significantly reduce voriconazole concentration. Methylprednisolone and dexamethasone reduce it to a greater extent than prednisone and prednisolone, and correlate with a higher glucocorticoid receptor potency noted for these two compounds. The in vitro study identified glucocorticoid receptor’s binding sites in the CYP2C19 gene. Dexamethasone upregulated the CYP2C19 promoter in HEPG2 cells, which proved its inductive effect [79]. In the in vivo study, the glucocorticoid-mediated induction resulted in an increased metabolism, which is the plausible mechanism that can explain the change [42]. The retrospective study conducted by Cojutti et al. [43] showed that coadministration with methylprednisolone, dexamethasone, rifampicin, phenobarbital, or carbamazepine led to a decrease in the voriconazole concentration. The statistical analysis showed that an increase of C_min_ for voriconazole caused by the coadministration with omeprazole and pantoprazole is completely counteracted when some CYP inducers are also introduced to the therapy. These findings confirmed the thesis drawn by Wang et al., that the coadministration of the inhibitor of CYP2C19 (omeprazole) and an inducer of CYP3A4 (dexamethasone) could decrease the potential effects of these medications [80]. The clinically relevant interaction between dexamethasone or prednisone and voriconazole was noted by Wallace et al. and Blanco-Dorado et al. [81,82]. In the former study, subtherapeutic concentrations of voriconazole were observed in a patient with a fungal brain abscess who concomitantly was treated with dexamethasone. The discontinuation of dexamethasone resulted in the elevation of the drug level. The latter study describes the patient with allergic bronchopulmonary aspergillosis. The discontinuation of prednisone but maintenance of voriconazole therapy resulted in photophobia, proximal myalgia, asthenia, and elevated bilirubin and liver enzymes. The concentration of voriconazole increased 5.6-fold after the withdrawal of the steroid. The discontinuation of the antifungal agent resulted in a withdrawal of the adverse side effects after a few days, and a recovery of liver parameters after two months [82]. These two reports proved that the therapeutic drug monitoring for voriconazole should be performed when glucocorticoid and voriconazole are concomitantly used.

#### 3.2.9. Oxycodone

Hagelberg et al. [83] investigated the effect of voriconazole on the pharmacokinetics of oxycodone. It inhibits CYP3A, CYP2C19, and CYP2C9, and there is a potential risk of interaction with oxycodone that undergoes the metabolism by CYP3A and CYP2D6. The statistical analysis showed that voriconazole increased ca. 1.7-fold the plasma concentration of oxycodone. The exposition on the drug also increased—the AUC_0-∞_ was higher ca. 3.6-fold. The elimination phase was prolonged—the half-life increased from 3.5 h to 7.1 h. Clearance and volume of distribution were reduced by 71% and 43%, respectively. The interaction also affected the metabolites noroxycodone, oxymorphone, and noroxymorphone. The coadministration of voriconazole led to a decrease of C_max_ of noroxycodone by 87% and AUC_0-∞_ by 67%. The two-fold prolongation was observed for the half-life. For the other metabolite—oxymorphone—a two-fold increase for C_max_ and a 7.3-fold increase of the average AUC_0-∞_ were observed. The elimination half-life changed from 3.6 h to 23.1 h after the administration of voriconazole. For noroxymorphone, the half-life prolonged ca. 3.7-fold. Changes in the pharmacokinetics are caused by the inhibition of CYP3A-mediated N-demethylation of oxycodone, which increased AUC_0-∞_. This resulted from the inhibition of the first pass metabolism and was also proven by the metabolite-to-oxycodone ratios. The increase of oxymorphone concentration after the administration of voriconazole resulted from the compensatory CYP2D6 metabolic route (O-demethylation). However, it could not replace it. The administration of voriconazole modestly increased the behavioral effects of heterophoria and miosis. The elevation in the concentration of oxycodone led to the increase of heat-pain and cold-pain threshold and decreased the mean AUC_0-12_ cold-pain intensity at 60 s. Side effects such as vomiting, and nausea occurred when oxycodone and voriconazole were coadministered. This implies that lower doses of analgesic oxycodone should be administered when antifungal treatment with voriconazole is conducted [83].

### 3.3. The Application of Voriconazole at Patients with SARS-CoV-2

Invasive fungal infections can be dangerous in patients with SARS-CoV-2. Voriconazole interacts with corticosteroids, sedative drugs, and remdesivir. Its use must be associated with the application of therapeutic drug monitoring due to the genetic polymorphism of CYP3A4 [84]. In COVID-19 treatment, some experimental therapies are involved. The interactions are observed for therapies with the following drugs: hydroxychloroquine, atanzavir, and lopinavir/ritonavir [85]. The other drug for which the potential interactions might be observed is azithromycin [86]. However, a previous study conducted on healthy volunteers proved that voriconazole does not affect the pharmacokinetics of azithromycin [54]. The other issue that must be taken into consideration is the prolongation of the QT-interval in patients treated with voriconazole. The risk is increased in patients with COVID-19 [86].

### 3.4. Conclusions

Vorioconazole absorption depends on the pH of the GI tract. High doses of PPI increase the trough concentration of the antifungal agent. Moreover, some PPI are also competitive inhibitors of CYP2C9, which make the interaction in in vitro tests possible. Voriconazole is a drug that is mainly metabolised by CYP2C19. Due to its hetergenicity it may lead to side effects. NSAID statins are the drugs that are widely used and their coadministration with voriconazole should be done with caution, as well for as herbal drugs. Due to the prolongation of QT-interval in patients treated with voriconazole it should be used cautiously in patients with COVID-19. Contrary to the other drugs presented in the manuscript, vorconazole does not have the affinity to P-gp. The interactions are listed inTable 2.

## 4. Posaconazole

Posaconazole is a representative of the second generation triazole antifungal agent with a broad spectrum of antifungal activity. It is indicated in the treatment of invasive fungal infections such as aspergillosis, fusariosis, and zygomycosis. In the European Union, it is approved to treat refractory IFI as a first-line agent in oropharyngeal candidiasis. In the US, it is used for the prophylaxis of *Aspergillus* and *Candida* infections refractory for itraconazole and/or fluconazole. The other indication for posaconazole therapy is oesophageal or febrile neutropenia, and the lack of effects or intolerance for other antifungal therapy [87,88]. It is available on the market in the solid formulation as tablets or in liquid form (as suspension) [10].

The therapeutic dose is 800 mg/day administered BID; however, the range is broad—it comprises 50–800 mg. A further increase in the serum concentration is not observed for the dose that exceeds 800 mg. Its bioavailability depends on the formulation. It increases for the suspension and the fed state [87,89]. The elimination half-life amounts to 31 h [90]. The high volume of distribution, 500 L, suggests extensive tissue distribution. The maximum concentration is reached after 3–5 h post administration, and the steady-state is reached after 7 to 10 days [88]. It has a strong affinity to proteins—it is bound in >95% [91].

### 4.1. Impact of Food and pH in the Gastrointestinal Tract on the Absorption of Posaconazole

The fasting state influences the bioavailability of posaconazole. Courtney et al. and Sansone-Parsons et al. [89,92] examined the impact of food and nutritional supplements on the pharmacokinetics of posaconazole in healthy patients. In the study conducted by Courtney et al. [89], a single oral dose of posaconazole 200 mg/5 mL in a suspension with both high-fat and non-fat breakfasts, and after 10 h fast. The tablets (2 × 100 mg) were administered with a high-fat meal. The analysis of the pharmacokinetic parameters showed that high-fat and non-fat meals enhanced the relative bioavailability of the posaconazole suspension over 290% and 168%, respectively. The absorption rate remained on the same level. The pharmacokinetic profiles for tablets given with fatty meals and the suspension given with non-fat meals were similar. The pharmacokinetic profile for a tablet given with a fatty meal was similar to the one given in a suspension with a non-fat meal. This implies that both coadministration with food regardless of the fat content, and in the form of suspension are the key factors that influence the exposure of the patient to the drug. The suspension is more convenient for patients that have troubles with swallowing [89]. Sansone-Parsons et al. examined the impact of nutrition supplements on the pharmacokinetic parameters of posaconazole. The coadministration of the drug suspension (400 mg/10 mL) with a nutrition supplement led to an increase of AUC and C_max_. The half-life and t_max_ remained on the same level. These studies indicated that posaconazole should be administered with meals, regardless of both the type of food (solid or liquid) and the fat content (non-fat vs. high fat) [89,92].

Antifungal agents such as ketoconazole and itraconazole are weak bases. Posaconazole is structurally related to itraconazole and has similar physicochemical properties. Carlson et al. investigated the solubility of ketoconazole in various pH, and found that it was more soluble under acidic conditions [93]. The pH reduced the absorption of ketoconazole in patients with achlorhydria (also related to AIDS) and those receiving H_2_-antagonist or antacids [94,95]. The simultaneous application of H_2_-antagonist with itraconazole also reduced the bioavailability of itraconazole [96]. Courtney et al. [97] investigated the impact of antacids on the pharmacokinetics of posaconazole under fasting and non-fasting conditions. Posaconazole is characterized by a high permeability and low aqueous solubility. Coadministration of the antacids did not have a statistically significant effect on posaconazole bioavailability, regardless of the fasting conditions—the absorption was not pH-dependent. For the fasting state, a 15% increase and a decrease of 12% in the nonfasting condition of the relative bioavailability were observed, but this was not considered as a clinically relevant change. The intersubject variability of AUC caused it. The other factor that influences bioavailability is the fasting state. The C_max_ of posaconazole was more than three-fold higher under non-fasting conditions, regardless of the administration of antacids. It also resulted in a ca. four-fold increase of AUC when compared with the fasting state. It also confirms the conclusions of the previous study where food improved the bioavailability of posaconazole. However, the rate of absorption, elimination, and t_max_ were not affected by antacids [89].

Krishna et al. [88] examined the effect of gastric pH (part 1), the dosing frequency and the prandial state (part 2), the timing of food relative to the time of posaconazole administration (part 3), and gastric motility (part 4). In part 1, a single dose of 400 mg posaconazole was administered concomitantly either with the acidic carbonated beverage or PPI (esomeprazole) or with an acidic carbonated beverage with PPI. The coadministration of posaconazole with the beverage increased the AUC and C_max_ by 70% and 92%, respectively. The situation changed when PPI was administered. The pH increase caused by PPI led to a decrease of posaconazole C_max_ and AUC by 46% and 32%, respectively. The simultaneous administration of posaconazole with PPI and acidic beverages also led to a decreased AUC and C_max_ by 21% and 33%, respectively. The half-life remained stable (25.2–27.8 h). The coadministration with esomeprazole reduced the intersubject variability. An abnormal pH level is often found in patients who require antifungal therapy, and acidic carbonated beverages may improve absorption. The opposite situation is in the case of PPI—with elevated pH and a decrease in the level of absorption. These findings are different from the previous study, where the impact of antacid was tested [97]. A slight increase in both C_max_ and t_max_ were observed. However, it was not statistically significant. This might be caused by the fact that in the former study, posaconazole was administered in the form of a tablet, whereas in the latter one it was administered in an oral suspension. The authors explained it with the fact that PPI has a much longer time of activity that causes an elevation of pH, contrary to inorganic antacids, which have a duration of action up to 3 h when administered with a meal [98]. The coadministration of posaconazole with an acidic beverage and esomeprazole resulted in the restoration of the AUC and C_max_ to the observed levels when administered alone under fasted conditions [88]. In part 2 of the study, the dosing regimen and the prandial state were investigated. The authors compared two dosing regimens in which the total dose of 800 mg was administered: posaconazole 400 mg BID and 200 mg QID for seven days. In both cases, they were given in a fasted state or with a nutritional supplement. The higher concentrations were observed when the drug was administered both with the nutritional supplement and when it was administered four times a day. The observed posaconazole levels and AUC were higher for QID than for BID, regardless of the fed state. The findings were similar to the trial results conducted by Sansone-Parsons et al., where the nutritional supplement improved the C_max_ of the drug [92]. A similar investigation was done by Ezzet et al. [99], who compared the three different dosing regimens for posaconazole: once-, twice-, and four-times a day under fasted conditions in healthy subjects. In this trial, it occurred that the most effective dosing regimens were BID and QID. However, higher concentrations and AUC were observed for QID. An increase in bioavailability of 98% was observed when posaconazole was administered BID and 220% when QID instead of a single dose. The absorption’s saturation might cause an increase in the drug concentration. This was observed for doses higher than 800 mg [90]. Posaconazole is characterized by a high lipophilicity and permeability, and low water solubility. The absorption might be restricted by the low solubility [88,100]. The conclusions from these three trials are that the drug administration two- or four-times a day provides effective therapy. The other factor that has an impact on drug exposure is meal timing. The administration of posaconazole during the meal and 20 min after the meal led to an increase of C_max_ and AUC above three times compared with the fasted state. The administration of posaconazole 5 min before the high-fat meal led to the increase of AUC and C_max_ when compared with the fasted state. However, the most significant increase was observed when it was administered with the meal or right after it. The last factor that may have an impact on the drug concentration is gastric motility. In the study of Krishna et al., the impact of loperamide (reduces the motility) and metoclopramide (increases the motility) was investigated. The coadministration of posaconazole with a prokinetic agent (metoclopramide) decreased the AUC and C_max_, however it was not clinically relevant due to the wide therapeutic index of posaconazole. In the case of coadministration with an antikinetic agent, the absorption of posaconazole was not affected—AUC increased by 11% and C_max_ decreased by 3% [88].

The previous papers mentioned the studies where the exposure on posaconazole was smaller for tablets in comparison with the oral suspension that was available on the market. Krishna et al. [101] conducted a study where three new solid formulations (two for tablets and one for capsule) were tested. The goal of the study was to achieve similar exposure on posaconazole as for the oral suspension. The tested formulation was designed to release the drug in the elevated pH of the small intestine. The single dose was 100 mg. The matrix used was hypromellose acetate succinate, which is sensitive to the changes of pH. It should result in better absorption—the matrix is highly soluble, which makes posaconazole release, and its presence in the intestinal fluid prevents the recrystallization of the drug. In this way, the larger part of the dose is absorbed [102]. The study was conducted for both fed and fasted conditions. In the fasted conditions, significantly higher AUC and C_max_ were observed for the new oral formulations than for the oral suspension. The oral suspension administration under the fed conditions increased the exposure to the drug. However, the values for AUC and C_max_ were still lower than those observed for tested solid formulation. The AUC and C_max_ for solid formulations under fasted and fed conditions were not markedly affected by food. This implies that that the new tested formulations can be taken regardless of the food. The new formulations would also change the dosing frequency from two to four times a day to once a day, which would be more convenient for patients. Kersemaekers et al. [102] evaluated the effect of a high-fat meal on the bioavailability of posaconazole in delayed-release tablets available on the market. A dose of 300 mg was administered once a day. The tested formulation matrix was based on the hypromellose succinate acetate, as in the previous study [101]. The study proved that high-fat meal modestly increased the AUC—by about 1.5-fold. C_max_ was higher ca. 1.15-fold, and the median t_max_ shifted from 5 to 6 h. The higher impact of food was noted for posaconazole in the oral suspension [89]. However, the differences in the impact of the fed state on posaconazole bioavailability were noted for these two studies [101,102]. It might be caused by the difference in the applied dose—for a higher one, the food effect could be more significant. Both trials suggest that posaconazole in tablets can be taken without regard for food, which can be more convenient for patients.

### 4.2. The Interaction with CYPs

Posaconazole is an inhibitor of CYP3A4, and the IC_50_ is 1.3 µM. It does not have an affinity towards CYP1A2, CYP2A6, CYP2C9, CYP2C19, and CYP2D6. The IC_50_ exceeded 300 µM for these enzymes. Posaconazole is not metabolized by cytochromes enzymes to a significant extent. It is not involved in DDI as voriconazole [99,103,104].

According to FDA data, posaconazole is contraindicated with the concomitant therapy with sirolimus. It results in nine-fold increase in sirolimus concentration, which results in toxicity. The coadministration of posaconazole in tablets of 400 mg BID with 2 mg of sirolimus resulted in the increase of both C_max_ and mean AUC by 572% and 788%, respectively. This was caused by the fact that sirolimus is metabolized by CYP3A4, and posaconazole is a strong inhibitor of that enzyme. If the cessation of posaconazole treatment was not possible, the dose of sirolimus should be reduced. The recommended change is 40% decrease of dose every 3 days [105,106].

When coadministered with pimozide and quinidine, it can result in the QTc prolongation and cases of “torsade de pointes”, which is also explained with the inhibition of CYP3A4. Posaconazole should be administered cautiously in patients with arrhythmia [106,107]. The inhibition of the CYP3A4 enzyme also reflects on the group of statins. Atorvastatin, lovastatin, and simvastatin are metabolized by CYP3A4. The inhibition of metabolism results in a higher concentration of the drugs, which may lead to rhabdomyolysis. Lipid-lowering drugs are intensively metabolized by CYP3A4. Posaconazole caused a 10-fold increase in simvastatin concentration [106,108,109].

### 4.3. Ethnicity

The other factor that may influence pharmacokinetics is ethnicity. Li et al. [110] investigated the pharmacokinetics of posaconazole in adult Chinese male and female subjects. The dose was 300 mg, and it was administered orally in fasted and fed states, and in a short infusion in a fasted state. After infusion, exposure to the drug was higher for Chinese volunteers than for Western subjects [111]. A two-fold increase in the posaconazole exposure was noted for Chinese volunteers when administered with food in tablet formulation. It was higher than in Western subjects noted by Kersemaekers [102]. The differences for AUC and C_max_ between the Chinese and Western population observed for the oral posaconazole in the fasted state were in favor Western subjects—they were slightly higher. The calculated bioavailability for posaconazole based on the results of the following trials [102,110,111] was similar for the fed state in both groups: 85% and 87% for Western and Chinese subjects, respectively. The bioavailability noted for the fasted state was different: 56% vs. 42% for Western and Chinese volunteers, respectively. The observed exposure in Chinese subjects was well tolerated and efficacious [110,111].

### 4.4. The Interaction with P-gp

Posaconazole can be both a substrate and inhibitor for P-gp. The IC_50_ of posaconazole is 3 µM [35]. Shumaker et al. [112] reported the interaction between digoxin and posaconazole. It resulted in the atrial fibrillation with a slow ventricula response. The inhibition of P-gp by posaconazole resulted in increased exposure of digoxin. The concomitant use of these drugs should be done with caution.

### 4.5. Conclusions

Posaconazole is an antifungal drug for which pharmacokinetic parameters, C_max_, and AUC that highly dependent on food, nutritional supplements, and pharmaceutical formulation. Posaconazole is an inhibitor of CYP3A4 and should be coadministered cautiously with the drugs metabolized by this enzyme. It may result in serious modification of dosing intervals, as in the case of sirolimus.

The interactions for posaconazole are listed in Table 3.

## 5. Isavuconazole

Isavuconazole is the most recent second-generation triazole antifungal agent, approved in 2015 by the Food and Drug Administration and the European Medicines Agency for the primary treatment of invasive aspergillosis and mucormycosis [113]. It is commercially available in both intravenous and oral formulations as the highly water-soluble prodrug isavuconazonium sulfate. After *iv* administration, the prodrug immediately undergoes (half-life less than a minute in vitro) hydrolysis by plasma esterases to the active component, isavuconazole, and produces an inactive cleavage product [114,115,116]. Human studies and animal models have described a high oral bioavailability of isavuconazole approaching 98%. The same dose is used for oral and *iv* administration. It was given at 200 mg once daily, following a loading dose of 200 mg every 8 h for the first 48 h. Maximum plasma concentration C_max_ was reached after 2–3 h of oral administration and after 1 h of *iv* administration [117,118]. Previous studies proved that isavuconazole administered in an *iv* or oral form demonstrates linear and dose-proportional pharmacokinetics with low inter- and intra-subject variability among healthy subjects. The coefficient of variation ranged from 10 to 43% for C_max_ and from 11 to 37% for AUC_24_ in a multiple-dose pharmacokinetics study [119]. Furthermore, isavuconazole pharmacokinetics studies in patients with invasive fungal diseases were conducted. In the multicenter SECURE trial, patients were randomized to receive either voriconazole or isavuconazole. It showed similar pharmacokinetics with a low intra-subject variability and narrow distributions of trough levels in the isavuconazole group [120,121]. Similar results were found in the trough levels of patients with renal failure [122,123]. This may suggest that the therapeutic drug monitoring (TDM) of isavuconazole is not routinely recommended.

### 5.1. Impact of Food and pH in the Gastrointestinal Tract on the Absorption of Isavuconazole

Unlike other triazoles, oral absorption of the drug is not significantly dependent on food intake, enabling isavuconazole to be taken with or without food [124,125]. However, the literature data are limited. To the best of our knowledge, only one report covers this aspect. Schmitt-Hoffmann et al. conducted two open-label, single-dose randomized crossover studies and one open-label, multiple-dose, parallel-group study in healthy volunteers to determine the potential impact of food and elevated gastric pH on isavuconazole absorption. For the food-effect study, on days 1 and 43, the subjects received a single dose of oral isavuconazole 400 mg during either a standardized, high-fat breakfast or following an overnight fast. No differences in the mean isavuconazole plasma concentrations were observed under fed and fasted conditions. The geometric least square mean ratios (fed/fasted) for the AUC_∞_ and C_max_ of isavuconazole were 110% and 92%, respectively. These data indicate that dosing with food had no effect on the exposure to isavuconazole. For the potential pH effect in this work, subjects were randomized to receive either isavuconazole alone or combination therapy of isavuconazole plus esomeprazole. The first group received an oral dose of isavuconazole (200 mg) three times a day (t.i.d) on days 1 and 2 and once per day on days 3–5. The second group received esomeprazole (40 mg) oral plus isavuconazole (200 mg), which was given immediately after esomeprazole dosing. Pharmacokinetics parameters incluing AUC_τ_, C_max_, and t_max_ were tested. Small increases in AUC and C_max_ were observed when isavuconazole was given in combination with esomeprazole rather than as a monotherapy. However, they were not considered to be clinically relevant. The geometric least square mean rations for AUC_τ_ and C_max_ were 108% and 105%, respectively. These findings provide evidence that the absorption of the agent is not influenced by the gastric pH, nor by the coadministration of PPI [126].

### 5.2. The Interaction with CYPs

Isavuconazole, as with other triazole antifungal agents, is associated with several clinically significant pharmacokinetics drug-drug interactions. Primarily, these drug interactions are facilitated between isavuconazole and drugs that are substrates, inhibitors, and inducers of CYP3A4/3A5 [127,128]. As isavuconazole is a relatively new azole agent, only a few randomized trials have examined its drug-drug interactions.

In vitro studies have demonstrated that isavuconazole is a substrate for CYP3A4 and CYP3A5, an inhibitor of CYP3A4, CYP2C8, CYP2C9, CYP2C19, CYP2D6, P-gp, BCRP, and human OCT2. Isavuconazole is also a week inducer of CYP3A4, CYP2C8, CYP2B6, and CYP2C19. However, in vivo studies have indicated that isavuconazole is a mild/moderate inhibitor of CYP3A4; a mild inducer of CYPB6; and does not affect the pharmacokinetics of substrates of CYP1A2, CYP2C8, CYP2C9, CYP2C19, and CYP2D6 [115]. The examples of the interactions are listed below.

#### 5.2.1. Ketoconazole, Lopinavir/Ritonavir

Isavuconazole is a substrate for CYP3A4, and concomitant use of isavuconazole with drugs that inhibit or induce this enzyme should be avoided. Townsend et al. evaluated the effect of isavuconazole in healthy adults. The inhibitor ketoconazole has been shown to affect the exposure of isavuconazole. Coadministration of isavuconazole with oral ketoconazole increased isavuconazole AUC and C_max_ by 422% and 9%, respectively [129,130]. Another strong inhibitor of CYP3A4, lopinavir/ritonavir, significantly increased the exposure of isavuconazole. The study by Yamazaki et al. was designed to establish the pharmacokinetics and safety impact of the coadministration of antiretroviral drugs with isavuconazole. Mean AUC and C_max_ of isavuconazole were 96% and 74% higher during coadministration with lopinavir/ritonavir compared with isavuconazole alone. In contrast, AUC and C_max_ of lopinavir were 27% and 23% lower, and mean AUC and C_max_ of ritonavir were 31% and 33% lower in the presence vs. absence of isavuconazole, respectively [113]. It was extraordinary that the observed exposure of itraconazole was not increased more than two-fold compared to previous studies with ketoconazole, while ritonavir was a stronger CYP3A4 inhibitor than ketoconazole. The differences between these results are explained in part by differences in study design. Additionally, the authors mentioned that the differences might be attributed to the fact that isavuconazole is also metabolized by CYP3A5, and CYP3A5 has been demonstrated to be inhibited by ketoconazole, but not by ritonavir. Given this, it is possible that inhibition of isavuconazole metabolism by ritonavir was partially compensated by CYP3A5. The authors suggested that isavuconazole may be safely coadministered with lopinavir/ritonavir. However, patients should be monitored for reduced antiviral efficacy to ensure adequate therapy.

#### 5.2.2. Rifampicin, Midazolam, Ethinyl Estradiol/Norethindrone

The coadministration of isavuconazole with strong CYP3A4 inducers such as rifampicin, rifabutin, carbamazepine, long-acting barbiturates, an St. John’s wort is contraindicated [116]. Clinical studies performed in healthy adults by Townsend et al. proved that the pharmacokinetics parameters of isavuconazole AUC and C_max_ were 90% and 75% lower during coadministration with rifampicin compared with isavuconazole alone, respectively. In the same study, pharmacokinetics interactions between isavuconazole and midazolam, and ethinyl estradiol/norethindrone were evaluated. Following coadministration, AUC increased 103% for midazolam, 8% for ethinyl estradiol, and 16% for norethindrone; C_max_ increased by 72%, 14%, and 6%, respectively [129]. It is well known that the simultaneous administration of rifampicin with triazole antifungal drugs can significantly reduce their plasma concentration and reduce their therapeutic efficacy. Concomitant administration is not recommended. The authors concluded that caution should be exercised with the concomitant use of isavuconazole and midazolam, and a possible dose of midazolam during therapy should be considered.

#### 5.2.3. Warfarin

Coadministration of isavuconazole with the substrates of CYP2C9 was analyzed. Because patients receiving isavuconazole therapy also require treatment with warfarin the potential pharmacokinetics and pharmacodynamics drug-drug interactions was determined. Desai et al., in a phase I trial evaluated in healthy adults, showed that coadministration with isavuconazole increased the AUC of S- and R-warfarin by 11% and 20%, respectively, and decreased the C_max_ of S- and R-warfarin levels by 12% and 7%, respectively. However, the pharmacodynamics of warfarin is not affected by coadministration with isavuconazole. The mean area under international normalized ratio curve and maximum the international normalized ratio were 4% lower in the presence vs. absence of isavuconazole [131]. The researchers concluded that coadministration with isavuconazole had no clinically relevant effects on warfarin pharmacokinetics. In addition, no unchanged pharmacokinetics parameters of isavuconazole were observed in this study. Similar results were found by Yamakazi and colleagues. They suggested that isavuconazole has a minimal effect on CYP2C9 and CYP2C19, and thus can be used with warfarin without dosage adjustment [132].

#### 5.2.4. Bupropion

Isavuconazole is a mild inducer of CYP2B6 and exposure of CYP2B6 substrates. Systemic exposure of bupropion was reduced (by 42%) during coadministration with isavuconazole. Caution is advised if isavuconazole is coadministered with CYP2B6 substrates with a narrow therapeutic range (e.g., efavirenz and cyclophosphamide) [132].

### 5.3. Immunosuppresive Agents

Concomitant administration of isavuconazole with immunosuppressive agents can lead to higher levels of these drugs. Groll et al., in phase I trials, examined the interaction between isavuconazole, and cyclosporine, mycophenolic acid, prednisolone, sirolimus, and tacrolimus in healthy subjects. It was found that coadministration with isavuconazole increased the area under the concentration-time curves of tacrolimus, sirolimus, and cyclosporine by 125%, 84%, and 29%, respectively, and the AUCs of mycophenolic acid and prednisolone by 35% and 8%, respectively. In addition, C_max_ of tacrolimus, sirolimus, and cyclosporine were 42%, 65%, and 6% higher, respectively; while C_max_ of mycophenolic acid and prednisolone were 11% and 4% lower, respectively. The authors concluded that the degree of interactions between isavuconazole and immunosuppressive agents is less than that reported for other triazole antifungal agents [133]. The coadministration of isavuconazole with tacrolimus was analyzed in other studies. Kim et al. reported a case study where significant increases in tacrolimus concentrations in a lung transplant patients were observed. In this study, tacrolimus dose was empirically reduced by 43% from 3.5 mg twice daily to 2 mg twice daily to maintain tacrolimus concentration within a target range of 6–8 ng/mL. Based on clinical observation, the authors recommended a reduction of the initial tacrolimus dose by 50% with the initiation of isavuconazole therapy, assuming the need for a further reduction of the tacrolimus dose by 25–50% [134]. Recent studies indicate different recommendations for the concomitant use of isavuconazole and tacrolimus. Rivosecchi et al. retrospectively evaluated the effect of isavuconazole on concurrent tacrolimus serum concentration in a group of 55 solid organ transplant patients. They reported a median 13% decrease in tacrolimus concentration/dose ratio (C/D) and a 1.3-fold increase in the daily dose of tacrolimus after isavuconazole was discontinued to maintain the required tacrolimus levels. The authors concluded that an empiric tacrolimus dose reduction is likely unnecessary of combination therapy with isavuconazole. Instead, tacrolimus TDM is recommended to individually guide tacrolimus dosing [135]. This finding appears to be similar to that reported by Kufel et al. In that study, potential drug-drug interactions with tacrolimus and isavuconazole were predicted in allogeneic stem cell transplant patient (alloSCT). A 40% of tacrolimus dose reduction was implemented based on previous recommendations provided by Kim et al. However, investigators concluded that empiric dose reduction was not necessary, as tacrolimus trough concentrations subsequently declined, requiring an increase in tacrolimus dose to maintain therapeutic trough concentrations. In addition, no affected isavuconazole trough concentration was documented. The measured steady-state drug level was 4 μg/mL, which was in line with the previously described studies [136]. The authors also recommended TDM of tacrolimus to guide drug dosing.

### 5.4. Isavuconazole with Transporters

As isavuconazole is used in immunocompromised patients with systemic mycoses who require multiple drugs concomitantly, clinical trials were performed to examine the possible interactions between isavuconazole and substrates of transporters. Yamazaki et al. conducted clinical trials to examine the potential DDI between itraconazole and atorvastatin, digoxin, metformin, and methotrexate in healthy human subjects [137]. This study proved that coadministration with isavuconazole resulted in approximately 37%, 25%, and 52% increases in the exposure of atorvastatin, digoxin, and metformin, respectively. An increase of C_max_ to 103%, 133%, and 123%, of atorvastatin, digoxin, and metformin, respectively, was also observed. Methotrexate parameters were unaffected by isavuconazole. The observed increases in plasma atorvastatin and digoxin concentrations, during coadministration with isavuconazole were significantly lower than that reported for other triazole antifungal agents. The therapeutic drug monitoring and adverse reactions of digoxin and atorvastatin during concomitant administration of isavuconazole are recommended by the authors.

The affinity of isavuconazole to P-gp was proven by Lempers et al. [35]. Isavuconazole inhibits P-gp at low micromolar concentrations—IC_50_ is 3 µM and is similar to itraconazole and posaconazole.

### 5.5. Conclusions

Isavuconazole is safer than voriconazole due to its linear pharmacokinetics. Studies conducted so far have shown that isavuconazole may be beneficial due to its limited drug-drug interactions compared to other triazole antifungal agents. However, concomitant medications with isavuconazole should be monitored and adjusted if necessary. Besides, isavuconazole does not affect the QTc prolongation, contrary to voriconazole and can be considered in the treatment of COVID-19 patients with complicated invasive pulmonary aspergillosis [86]. The interactions are listed in Table 4.

## 6. Summary

The effectiveness of the treatment of fungal infections with azoles is influenced by factors such as the type of food, the pH of the GI-tract, and the concomitant use of other drugs. In the case of the second generation of azoles, the impact of food might vary between the drugs. For itraconazole it is unpredictable. However, taken with a meal or shortly after it may improve the bioavailability. Posaconazole is a drug for which the bioavailability is increased when taken with meals (especially the high fat meals) and dietary supplements. The absorption of isavuconazole is not influenced by food intake. It can be administered regardless of the meal. On the other hand, voriconazole should not be taken with a meal.

The other factor that can modify the absorption is taking PPI. The acidic pH is required for the absorption of itraconazole. A similar situation is observed for posaconazole. The elevation in pH reduces bioavailability. In the case of isavuconazole, absorption is not affected by PPI. The co-administration did not lead to statistically significant changes.

The other interaction that may have an impact on therapy is the interaction with CYP enzymes. The most significant is the interaction with CYP3A4, observed for itraconazole, voriconazole, and posaconazole. They are the inhibitors of this enzyme. The antifungal drugs can interact with other enzymes such as CYP2B6 (isavuconazole) and CYP2C9 (voriconazole). Itraconazole is metabolized mainly by CYP3A4, whereas voriconazole by CYP2C19. Posaconazole is not metabolized by cytochromes to a significant extent. It might be considered as a potentially safer drug.

P-gp is a transporter that removes xenobiotics from the body. The clinical trials and in vitro studies have proven that itraconazole, posaconazole, and isavuconazole are the inhibitors of P-gp. Voriconazole has not an affinity to that transporter. This is a significant issue when the other drugs that are substrates for P-gp are co-administered. The complexity of interactions may lead to the lack of fungicidal effect and failure of the treatment, therefore the therapy of the patients with use of azoles should be supported with therapeutic drug monitoring.

## Figures and Tables

**Table 1:** The interactions for itraconazole with drugs.

Drug	The Impact of pH on Absorption	Reference
Omeprazole	Decrease of AUC_0-24_ and C_max_ of itraconazole.	[18]
	**The Impact of Interaction with CYP on Pharmacokinetic Parameters**	
Ibrutinib	The inhibition of CYP3A4 by itraconazole results in 10-fold increase of AUC and 8.8-fold increase of C_max_ for ibrutinib.	[22]
Efavirenz	The induction of CYP3A4 activity by efavirenz led to decrease of exposure to itraconazole and its metabolite.	[24]
Atorvasatatin	The inhibition of CYP3A4 by itraconazole resulted in the increase in AUC, the peak serum concentration and half-life of atorvastatin lactone.	[26]
Oxycodone	The inhibition of the gut and liver CYP3A4 resulted in the increase in the exposure to oxycodone via inhibition of N-demetylation.	[28]
Midazolam	The inhibition of CYP3A4 by itraconazole resulted in the increase in the concentration of midazolam.	[29]
Alprazolam	The inhibition of CYP3A4 by itraconazole resulted in the increase in AUC and prolongation of half-life of alprazolam and decrease in the oral clearance.	[30]
Haloperidol	Voriconazole increased of AUC of haloperidol for both genetic types—CYP2D6*10/*10 and CYP2D6*1/*1.	[31]
Phenytoin	The inhibition of CYP2C9 by itraconazole increased the AUC of phenytoin by 10%.	[32]

**Table 2:** The interactions for voriconazole with drugs.

Drug	The Impact of pH on Absorption	Reference
Raniditine and pantoprazole	Ranitidine decreased the trough voriconazole concentration; pantoprazole increased the trough concentration.	[40]
	**The Impact of Interaction with CYP on Pharmacokinetic Parameters**	
Ritonavir	Ritonavir inhibits CYP3A4 which leads to increase of exposure to voriconazole	[45]
Ibuprofen	The inhibition of CYP2C9 by voriconazole resulted in the increase in the S-(+) ibuprofen AUC and C_max_. Weak effect on pharmacokinetics of R-(−) ibuprofen.	[56]
Imatinib	Voriconazole inhibits CYP3A4 which results in increase of C_max_ of imatinib.	[62]
St John’s wort	The interaction of hyperforin with CYP2C9 resulted in short term but clinically irrelevant increase of AUC followed by a prolonged extensive reduction in voriconazole exposure.	[70]
Simvastatin	Case study. Voriconazole inhibited CYP3A4 related metabolism of simvastatin. The rhabdomyolisis occurred and patient died of respiratory failure.	[76]
Rifampicin	Rifampicin induced CYP3A4 which led to the decrease in voriconazole C_max_ and AUC_τ_.	[77]
Oxycodone	Increase of AUC, C_max_, and elimination half-life of oxycodone due to the inhibition of CYP3A by voriconazole.	[83]
	**The Impact of Glucocorticoids**	
Prednisone	Case study. The discontinuation of prednisone resulted in adverse side effects of voriconazole which were the results of the elevation of the anti-fungal agent level after steroid withdrawal.	[82]
Dexamethasone	Case study. The concomitant treatment voriconazole and dexamethasone resulted in a low levels of voriconazole.	[81]

**Table 3:** The interactions of posaconazole.

Type of Interaction	The Result of Interaction	Reference
The impact of food and formulation on the relative bioavailability	Food enhanced the absorption of posaconazole and the higher bioavailability is observed for the suspension.	[89]
The impact of nutritional supplements on the posaconazole bioavailability	Nutrition supplement increased the C_max_ and AUC.	[92]
Antacids	No statistically significant impact on posaconazole bioavailability under fasting or nonfasting conditions.	[97]
Gastric pH, dosing regimen, meal timing, and effect of gastric motility on the absorption	The acidic carbonated beverage increased the C_max_ and AUC of posaconazole. The coadministration with PPI led to decrease of C_max_ and AUC. The dose should be divided into 2 or 4 doses a day.	[88]
Impact of the new solid formulation on the exposure	The tested formulations had a better bioavailability than oral suspension regardless the meal.	[101]
The impact of high-fat meal	Exposure of posaconazole administered in delayed-release tablets is modestly affected by a high-fat meal but it can be given in tablet either in fast or fed state.	[102]
The evaluation of the pharmacokinetics and safety of the therapy in the Chinese subjects	Higher exposition on posaconazole after *iv* administration for Chinese subjects. The relative bioavailability for the fed state in Chinese subjects was similar to the Western subjects.	[110]

**Table 4:** The different interactions of isavuconazole.

Subject of Interaction	The Impact of pH on Absorption	Reference
The effect of food and pH on the absorption	The absorption was not affected.	[126]
	**The Impact of Interaction with CYP on Pharmacokinetic** **Parameters**	
Cyclosporine, mycophenolic acid, prednisolone, sirolimus, and tacrolimus	Increase in AUC and C_max_ of immunosuppressive agents.	[133]
Tacrolimus	The interaction is most significant following liver transplantation. Case study. An initial 50% reduction and TDM of tacrolimus concentration ware recommended.	[134,135]
Rifampicin, ketoconazole and midazolam	Rifampicin induced CYP3A4 which led to the decrease in isavuconazole AUC.Exposure of isavuconazole increased 4.2-fold by ketoconazole. Exposure increased 103% of midazolam.	[129]
Atorvastatin, digoxin and metformin	The inhibition of CYP3A4 by isavuconazole resulted in the increase inAUC of atorvastatin, digoxin, and metformin.	[137]
Lopinavir/ritonavir	The AUC of isavuconazole increses by-2-fold with strong inhibitor of CYP3A4. The AUC of lopinavir and ritonavir were 27% and 31% lower, respectively, in the presence of isavuconazole.	[113]
Bupropion	The induction of CYP2B6 activity by isavuconazole led to reduce of bupropion exposure (by 42%)	[132]

## Data Availability

Not applicable.
